# DotU expression is highly induced during *in vivo* infection and responsible for virulence and Hcp1 secretion in avian pathogenic *Escherichia coli*

**DOI:** 10.3389/fmicb.2014.00588

**Published:** 2014-11-07

**Authors:** Shaohui Wang, Jianjun Dai, Qingmei Meng, Xiangan Han, Yue Han, Yichao Zhao, Denghui Yang, Chan Ding, Shengqing Yu

**Affiliations:** ^1^Shanghai Veterinary Research Institute, Chinese Academy of Agricultural SciencesShanghai, China; ^2^College of Veterinary Medicine, Nanjing Agricultural UniversityNanjing, China

**Keywords:** avian pathogenic *Escherichia coli*, type VI secretion system, DotU, secretion, virulence

## Abstract

Type VI secretion systems (T6SSs) contribute to pathogenicity in many pathogenic bacteria. Three distinguishable T6SS loci have been discovered in avian pathogenic *Escherichia coli* (APEC). The sequence of APEC T6SS2 locus is highly similar to the sequence of the newborn meningitis *Escherichia coli* (NMEC) RS218 T6SS locus, which might contribute to meningitis pathogenesis. However, little is known about the function of APEC T6SS2. We showed that the APEC T6SS2 component organelle trafficking protein (DotU) could elicit antibodies in infected ducks, suggesting that DotU might be involved in APEC pathogenicity. To investigate DotU in APEC pathogenesis, mutant and complemented strains were constructed and characterized. Inactivation of the APEC *dotU* gene attenuated virulence in ducks, diminished resistance to normal duck serum, and reduced survival in macrophage cells and ducks. Furthermore, deletion of the *dotU* gene abolished hemolysin-coregulated protein (Hcp) 1 secretion, leading to decreased interleukin (IL)-6 and IL-8 gene expression in HD-11 chicken macrophages. These functions were restored for the complementation strain. Our results demonstrated that DotU plays key roles in the APEC pathogenesis, Hcp1 secretion, and intracellular host response modulation.

## INTRODUCTION

The protein secretion system is a common strategy for successful infection in hosts by Gram-negative bacteria (Saier, 2006). The type VI secretion system (T6SS) was first discovered in *Vibrio cholerae* (*V. cholerae*) in 2006 (Pukatzki et al., 2006; Records, 2011) and has been identified in more than one-fourth of all sequenced bacterial genomes (Filloux et al., 2008; Shrivastava and Mande, 2008; Boyer et al., 2009; Filloux, 2009). The T6SS contributes to the pathogenicity of many bacteria (Zheng and Leung, 2007; de Pace et al., 2010; Miyata et al., 2010; Mulder et al., 2012; Rosales-Reyes et al., 2012; Zhou et al., 2012; Ho et al., 2014) and to bacteria–host interactions or interbacterial interactions in non-pathogenic bacteria (Jani and Cotter, 2010; Basler et al., 2013).

Although the exact function of most T6SS proteins is not known, the majority are necessary for secretion of effector proteins including hemolysin-coregulated protein (Hcp), valine-glycine repeat protein (VgrG), ClpV, intracellular multiplication protein (IcmF) and organelle trafficking protein (DotU; Wu et al., 2008; Pukatzki et al., 2009). Hcp and VgrG are mutually dependent for secretion in *V. cholerae*, *Edwardsiella tarda* and enteroaggregative *Escherichia coli* (EAEC; Dudley et al., 2006; Pukatzki et al., 2007; Zheng and Leung, 2007), suggesting that Hcp and VgrG are secreted proteins and machine components. ClpV energizes secretion of effector proteins that form oligomeric complexes that enable ATP hydrolysis-dependent protein transport (Schlieker et al., 2005; Pukatzki et al., 2009). IcmF is a component of the T6SS apparatus that is required for secretion by the T6SS and intracellular growth during infection. DotU stabilizes the secretion machinery and was essential for the intracellular life cycle and virulence of *Francisella tularensis* (Sexton et al., 2004; Zusman et al., 2004; Broms et al., 2012).

Systemic infections caused by avian pathogenic *Escherichia coli* (APEC) are economically devastating to poultry industries (Rodriguez-Siek et al., 2005b; Ewers et al., 2007). Moreover, APEC has a broad range of virulence factors similar to uropathogenic *Escherichia coli* (UPEC) and newborn meningitis *Escherichia coli* (NMEC), indicating that APEC may be a potential virulence gene reservoir for UPEC and NMEC (Wang and Kim, 2002; Rodriguez-Siek et al., 2005a; Moulin-Schouleur et al., 2006; Ewers et al., 2007; Johnson et al., 2008; Tivendale et al., 2010; Wang et al., 2011a). Three distinct and conserved T6SS loci, T6SS1, T6SS2 and T6SS3, are present in APEC genomes. T6SS1 and T6SS3 in APEC have homologs in EAEC. The T6SS2 in APEC is similar to the T6SS in NMEC RS218 (Ma et al., 2013). The T6SS1 core components (ClpV and Hcp) in the APEC strain SEPT362 are involved in adherence to and actin rearrangement in epithelial cells but are not involved in intramacrophage replication. The T6SS2 core components (Hcps) in NMEC RS218 coordinately function in steps of RS218 interaction with human brain microvascular endothelial cells (HBMECs) such as binding to and invasion of HBMECs, cytokine and chemokine release, and apoptosis. The T6SS3 locus lacks several key genes and is non-functional (de Pace et al., 2010; Zhou et al., 2012). However, the function of T6SS2 core genes in APEC remains unknown.

In this study, the *dotU*-inactivated mutant and complementation strains were constructed from APEC strain DE719. The effects of DotU on normal duck serum resistance, cytopathogenicity, intramacrophage survival, Hcp1 secretion, and virulence were investigated.

## MATERIALS AND METHODS

### BACTERIAL STRAINS, PLASMIDS, AND GROWTH CONDITIONS

Strains and plasmids are shown in **Table 1**. The wild-type APEC strain DE719 was isolated from a duck with clinical septicemia symptoms of colibacillosis in Jiangsu, China and identified as an APEC strain by phenotypic characters and virulence genes presence (Ewers et al., 2005). The serotype was identified by agglutination test with rabbit anti-*Escherichia coli* immune serum (Statens Serum Institut, Copenhagen, Denmark) and allele-specific PCR (Wang et al., 2014). Infection studies confirmed that APEC DE719 caused severe colibacillosis symptoms and high mortality in ducks and mice. *Escherichia coli* strain DH5α was used for cloning and strain BL21 (DE3) was used for protein expression (Davanloo et al., 1984; Studier and Moffatt, 1986). All *Escherichia coli* strains were grown in Luria-Bertani (LB) medium at 37°C with aeration. When necessary, medium was supplemented with ampicillin (Amp; 100 μg/mL) or chloramphenicol (Cm; 30 μg/mL).

**Table 1:** Bacterial strains and plasmids used in this study.

Strains or plasmids	Characteristics	Reference
**Strain**
DE719	O2:K1	
DE719ΔdotU	*dotU* deletion mutant in DE719	This study
DE719CΔdotU	DE719Δ*dotU* with plasmid pSTV28-dotU	This study
DE719ΔT6SS2	T6SS2 deletion mutant in DE719	This study
DE719Δhcp1	*hcp1* deletion mutant in DE719	This study
DH5α	F –, *Δ(lacZYA-argF)U169, recA1, endA1, hsdR17(rk–, mk+), phoA, supE44, λ–*	TIANGEN
BL21 (DE3)	F –, *ompT, hsdS (r_B_^-^ m_B_^-^) gal, dcm* (DE3)	TIANGEN
**Plasmid**
pET28a(+)	Kan, F1 origin, His tag	Novagen
pET28a-dotU	pET28a (+) carrying *dotU* gene	This study
pET28a-hcp1	pET28a (+) carrying *hcp1* gene	This study
pET28a-hcp2	pET28a (+) carrying *hcp2* gene	This study
pMD 18-T Vector	Amp, lacZ	Takara
pSTV28	Cm, lacZ	Takara
pSTV28-dotU	pSTV28 derivative harboring dotU	This study
pKD46	Amp; expresses λ red recombinase	Datsenko and Wanner (2000)
pKD3	Cm gene, template plasmid	Datsenko and Wanner (2000)
pCP20	Cm, Amp, yeast Flp recombinase gene, FLP	Datsenko and Wanner (2000)

### EXPRESSION AND PURIFICATION OF RECOMBINANT DotU, Hcp1, AND Hcp2 PROTEINS

DNA manipulation and transformation were performed using standard methods. All restriction enzymes were purchased from TaKaRa (Dalian, China). Plasmid DNA was isolated using High Pure Plasmid Miniprep kits (Invitrogen, San Diego, CA, USA). PCR product purification and DNA extractions from agarose gels used Agarose Gel DNA Fragment Recovery Kits (TaKaRa) according to the manufacturer’s guidelines. Open reading frames (ORFs) of *dotU*, *hcp1,* and *hcp2* were amplified with primers in **Table 2** and subcloned into pET28a (+) vector (Novagen, Madison, WI, USA). Recombinant plasmids were transformed into competent *Escherichia coli* BL21 (DE3) and proteins were expressed by isopropyl-beta-D-thiogalactopyranoside (IPTG) induction at a final concentration of 1 mM. Fusion proteins were purified using HisTrap HP columns (GE Healthcare, Shanghai, China) according to the manufacturer’s guidelines. Final protein concentrations were determined by Bradford method using SmartSpec3000 (Bio-Rad). Polyclonal antibodies were produced in New Zealand White rabbits as described previously (Dai et al., 2010; Wang et al., 2011a, 2012).

**Table 2:** Primers used in this study.

Primers	Sequence (5′–3′)^a^	Target genes
dotUEx-F	GAGGGATCCATGAGCGATATGAGTGAA	*dotU*
dotUEx-R	GACAAGCTTTTATCGGAGTAATTTATTGA	*dotU*
hcp1Ex-F	GCGGATCCAGCAAAATGAACAACAAT	*hcp1*
hcp1Ex-R	GTGCTCGAGTTTCTGAACGGCGATACC	*hcp1*
hcp2Ex-F	GCGGATCCCCAACCCCATGTTACATT	*hcp2*
hcp2Ex-R	GTGCTCGAGTGCTTCCAGCGGTGCACGCC	*hcp2*
dotUMu-F	AGCTTCCCCGATCTGAACCTCCAGCTCTGGGCTATAAGGGGATAAGTGAGTGTAGGCTGGAGCTGCTTC	pKD3
dotUMu-R	TAAGCCGCTTTACGAGCGTGGCTAAATCAATCTGGATCATAAGATGTCCCATATGAATATCCTCCTTAG	pKD3
T6SS2Mu-F	TTGCCTTTTTAAAATATAACAATAATGCAGATGAAAGACTCCCTGGTAACGTGTAGGCTGGAGCTGCTTC	pKD3
T6SS2Mu-R	GATTCACAGGCGTATAAAGCAAATACAATCACCATGTTTTATATCCTGCACATATGAATATCCTCCTTAG	pKD3
Hcp1Mu-F	GCAGTACGAAAATGCTGTGCTCATGGCCTGAACGGGAACATTTTTATGGTGTAGGCTGGAGCTGCTTC	pKD3
Hcp1Mu-R	GCAATTTCTTCCTTTACTGACATACTGAATATCCTTCTGTGAAAATTACATATGAATATCCTCCTTAG	pKD3
C1	TTATACGCAAGGCGACAAGG	pKD3
C2	GATCTTCCGTCACAGGTAGG	pKD3
dotUup-F	CTGGGAGAACTGATGACC	Upstream region of *dotU*
dotUdown-R	ACGTCCACCGGGATAACT	Downstream region of *dotU*
T6SS2up-F	GACTGACACGATGTCACTG	Upstream region of T6SS2
T6SS2down-R	CTTTTCACGCCATACTTC	Downstream region of T6SS2
Hcp1up-F	TGAAAGCACCGGCAGTGATG	Upstream region of *Hcp1*
Hcp1down-R	ACAGGGTTTTCATCCGGTGAG	Downstream region of *Hcp1*
chβactin-F	GAGAAATTGTGCGTGACATCA	β*-actin*
chβactin-R	CCTGAACCTCTCATTGCCA	β-*actin*
chIL6-F	GTTCGCCTTTCAGACCTAC	*IL-6*
chIL6-R	ACCACTTCATCGGGATTTA	*IL-6*
chIL8-F	TTGGAAGCCACTTCAGTCAGAC	*IL-8*
chIL8-R	GGAGCAGGAGGAATTACCAGTT	*IL-8*

*^a^Restriction sites are underlined.*

### CONSTRUCTION OF MUTANT AND COMPLEMENTATION STRAINS

The isogenic mutants DE719ΔdotU and DE719ΔT6SS2 were constructed according to the method of Datsenko and Wanner (2000). Chloramphenicol resistance cassettes flanked by upstream and downstream sequences of *dotU* or the T6SS2 locus were amplified and transformed into the APEC DE719-containing lambda red recombinase expression plasmid pKD46. After electroporation, samples were incubated at 37°C for 1 h in super optimal broth with catabolite repression broth and plated on LB agar with chloramphenicol. Resistant mutants were confirmed by PCR amplification and sequence analysis using primers C1 and C2 (Datsenko and Wanner, 2000) combined with primers flanking the *dotU* or T6SS2 region. The chloramphenicol resistance cassette was cured by transforming with plasmid pCP20 and selecting for chloramphenicol sensitive strain.

The upstream region of the *dotU* ORF contained no promoter, so the *dotU* gene was complemented in *trans* by cloning into plasmid pSTV28 using primers dotUEx-F and dotUEx-R (**Table 2**). The resulting plasmid pSTV28-dotU was transformed into DE719ΔdotU to generate strain DE719CΔdotU. The complementation strain was identified by PCR. To detect the effect of DotU on growth rate, growth kinetics of strains were determined.

### DUCK SERA PREPARATION AND ENZYME-LINKED IMMUNOSORBENT ASSAYS

To investigate if anti-DotU antibody was elicited during APEC infection, APEC DE719-infected duck sera, DE719ΔdotU pre-adsorbed anti-DE719 sera and inactivated APEC DE719-immunized duck sera were titered using an enzyme-linked immunosorbent assay (ELISA). APEC DE719-infected duck sera were produced as described previously (Zhuge et al., 2013). Ducks were infected intratracheally with live APEC DE719 at 5 × 10^6^ colony forming units (CFUs) twice over a 2-week interval. At 10 days after the second infection, serum was collected from the survivors. Pre-adsorbed APEC DE719-infected duck sera were prepared by adsorption to DE719ΔdotU bacteria at 37°C for 2 h. Immunized duck sera were obtained from 8 ducks vaccinated twice with ISA 71VG (Seppic, France) emulsified with formalin-inactivated APEC DE719 cells. Negative sera were obtained from 8 ducks inoculated with phosphate-buffered saline (PBS). Microtiter plates were overnight coated at 4°C with purified recombinant DotU at 0.5 μg/well. Wells were washed twice with PBST (PBS with 0.05% Tween-20) and blocked with PBST-5% skim milk for 1 h. After washing with PBST, duck antisera were 2-fold serial diluted, starting at 1:8 and added to ELISA plate wells for 2 h. Wells were washed three times and horseradish peroxidase (HRP)-conjugated anti-duck IgG (KPL, Gaithersburg, MD, USA) was used as the secondary antibody. Antibody against DotU was visualized by adding 100 μL 3,3′,5,5′-tetramethyl benzidine (Tiangen, Beijing, China), stopping with 100 μL 2 M H_2_SO_4_. Absorbance at 450 nm was determined with a plate reader (Bio-Tek Instruments, Winooski, VT, USA). Titers were defined as the reciprocal of the highest dilution of serum producing a 2.1-fold ratio value above negative serum.

### PREPARATION AND ANALYSIS OF SECRETORY PROTEINS

Secretory proteins were prepared as described previously with modifications (Zhou et al., 2012). Overnight bacterial cultures were diluted 1:100 into fresh LB medium and grown to logarithemic phase at 37°C with shaking. Bacteria were harvested and centrifuged at 10,000 × *g* for 15 min at 4°C. Supernatants were collected and filtered through a 0.22 μm membrane to remove bacterial cell contamination. Secretory proteins were precipitated from the supernatant using 10% trichloroacetic acid and washed with acetone. Secretory proteins quality was verified by Western blotting for the absence of the cytosolic marker cAMP receptor protein (CRP) using anti-CRP antibody (Santa Cruz, CA, USA).

Secretory proteins were analyzed as described (Wang et al., 2011a,b). Briefly, protein samples were subjected to sodium dodecyl sulfate-polyacrylamide gel electrophoresis (SDS-PAGE) and transferred onto a polyvinylidene fluoride membrane (Amersham Pharmacia Biotech, Piscataway, NJ, USA). Anti-Hcp1, anti-Hcp2 or anti-CRP antibody were used as primary antibodies. IRDye 800CW-conjugated donkey anti-rabbit polyclonal antibody (LI-COR) was the secondary antibody. Blots were visualized with the Odyssey Two-Color Infrared Imaging System (LI-COR).

### BACTERIAL RESISTANCE TO NORMAL DUCK SERUM

Normal duck serum was obtained from healthy 10-day-old Cherry-Valley ducks. No APEC antibodies were detected using ELISA. Bactericidal assays were performed in 96-well plates as described previously with some modifications (Gao et al., 2013). Briefly, normal duck serum was diluted to 5, 12.5, 25, and 50% in PBS. Bacteria were added to sera at different dilutions, incubated at 37°C for 30 min. Then, bacteria were enumerated by plating on LB agar plates. Heat-inactivated normal duck serum was used as a control.

### BACTERIAL ADHESION AND INVASION ASSAYS

Bacterial adhesion and invasion assays were as described previously (Wang et al., 2011a; Zhuge et al., 2013). Chicken embryo fibroblast DF-1 cell monolayers were washed with Dulbecco’s modiszfied Eagle’s medium (DMEM) without fetal bovine serum (FBS) and infected with bacteria at a multiplicity of infection (MOI) of 100 for 2 h at 37°C under 5% CO_2_. After washing with PBS, cells were lysed with 0.5% Triton X-100 and bacteria were counted by plating on LB agar plates. For invasion assays, cell cultures, bacterial infection, and bacterial counting were as described for bacterial adhesion assays. Cells were treated with DMEM containing gentamicin (100 μg/mL) for 1 h to kill extracellular bacteria. Monolayers were washed and lysed with 0.5% Triton X-100. Released bacteria were counted by plating on LB agar plates. Negative control wells containing DF-1 cells only were used in all experiments. Assays were performed three times in triplicate.

### INTRACELLULAR SURVIVAL ASSAYS

To determine bacterial intracellular survival capacity, chicken macrophage HD-11 cells were infected with bacteria as described for invasion assays. After 1 h of infection, cells were washed and treated with DMEM containing gentamicin (100 μg/mL) for 1 h to kill extracellular bacteria. Released bacteria were defined as bacteria initially invasive to HD-11 cells. To determine intracellular survival, cells were grown in DMEM containing 10 μg/mL gentamicin for 6, 12, or 24 h before lysis of cultured cells. Intracellular survival was expressed as change (*n*-fold) in bacterial number at a given time point relative to initial invasive bacteria.

### ANIMAL EXPERIMENTS

Animal experiments were carried out in accordance with guidelines of the Association for Assessment and Accreditation of Laboratory Animal Care International (AAALAC). The animal study protocol (13-05) was approved by the Animal Care and Use Committee of the Shanghai Veterinary Research Institute, Chinese Academy of Agricultural Sciences (CAAS), China.

To determine the effect of *dotU* on bacterial virulence, groups of eight 7-day-old ducks were inoculated intratracheally with bacterial suspensions of DE719, DE719ΔdotU, or DE719CΔdotU at 10^7^ CFU. Ducks inoculated intratracheally with PBS were used as negative controls. Mortality was monitored daily until 7 days after infection. Experiments were repeated three times.

Bacterial colonization was determined during systemic infections as described previously (Antao et al., 2009; Wang et al., 2011a,b). Briefly, groups of eight 7-day-old ducks were infected intratracheally with a bacterial suspension containing 10^8^ CFUs. At 24 h after infection, ducks were euthanized and dissected. Organs were homogenized, diluted and plated onto LB agar to determine bacterial numbers.

### QUANTITATION OF CYTOKINES EXPRESSION LEVELS IN HD-11 CELLS

The mRNA levels of cellular inflammatory cytokines in HD-11 cells infected with APEC strains were investigated by quantitative real-time reverse transcription PCR (qRT-PCR). In brief, total RNA was isolated from bacteria infected HD-11 cells using TRIZol®; reagent (Invitrogen). Contaminating DNA was removed from the samples with RNase-free DNase I (TaKaRa). cDNA synthesis was performed using the PrimeScript®; RT reagent kit (TaKaRa) according to the manufacturer’s protocol. qRT-PCR was performed using SYBR®; *Premix Ex* Taq^TM^ (TaKaRa) and gene-specific primers (**Table 2**). The relative gene expression was normalized to the β*-actin* gene via the ΔΔCt method (Livak and Schmittgen, 2001). All samples were calibrated to levels of gene expression of DE719 infected HD-11 cells. PCR efficiency (>90%) for each of the genes was verified via standard dilution curves. The assay was performed in duplicate and repeated three times.

### STATISTICAL ANALYSES

Statistical analyses used the GraphPad Software package (GraphPad Software, La Jolla, CA, USA). One-way analysis of variance (ANOVA) was used for analysis of adhesion assay data, and two-way ANOVA was performed for survival assays and qRT-PCR results. Animal infection data were analyzed using the non-parametric Mann–Whitney U-test. Survival curves were created by the Kaplan–Meier method using the product limit method and compared by the log-rank (Mantel–Cox) test. Figures show mean values. Statistical significance was established at *p* < 0.05.

## RESULTS

### DELETION OF *dotU* AND T6SS2 DOES NOT AFFECT GROWTH KINETICS AND APEC DE719 SWARMING

The mutant strains DE719ΔdotU, DE719ΔT6SS2, and complementation strain DE719CΔdotU were generated and confirmed by PCR. No significant growth defect was observed among them during growth in LB medium (data not shown). DE719ΔdotU and DE719ΔT6SS2 mutants migration was similar to the wild-type DE719 strain on swarming agar plates, indicating that motility was not affected by disruption of DotU and T6SS2 (data not shown).

**FIGURE 1:**
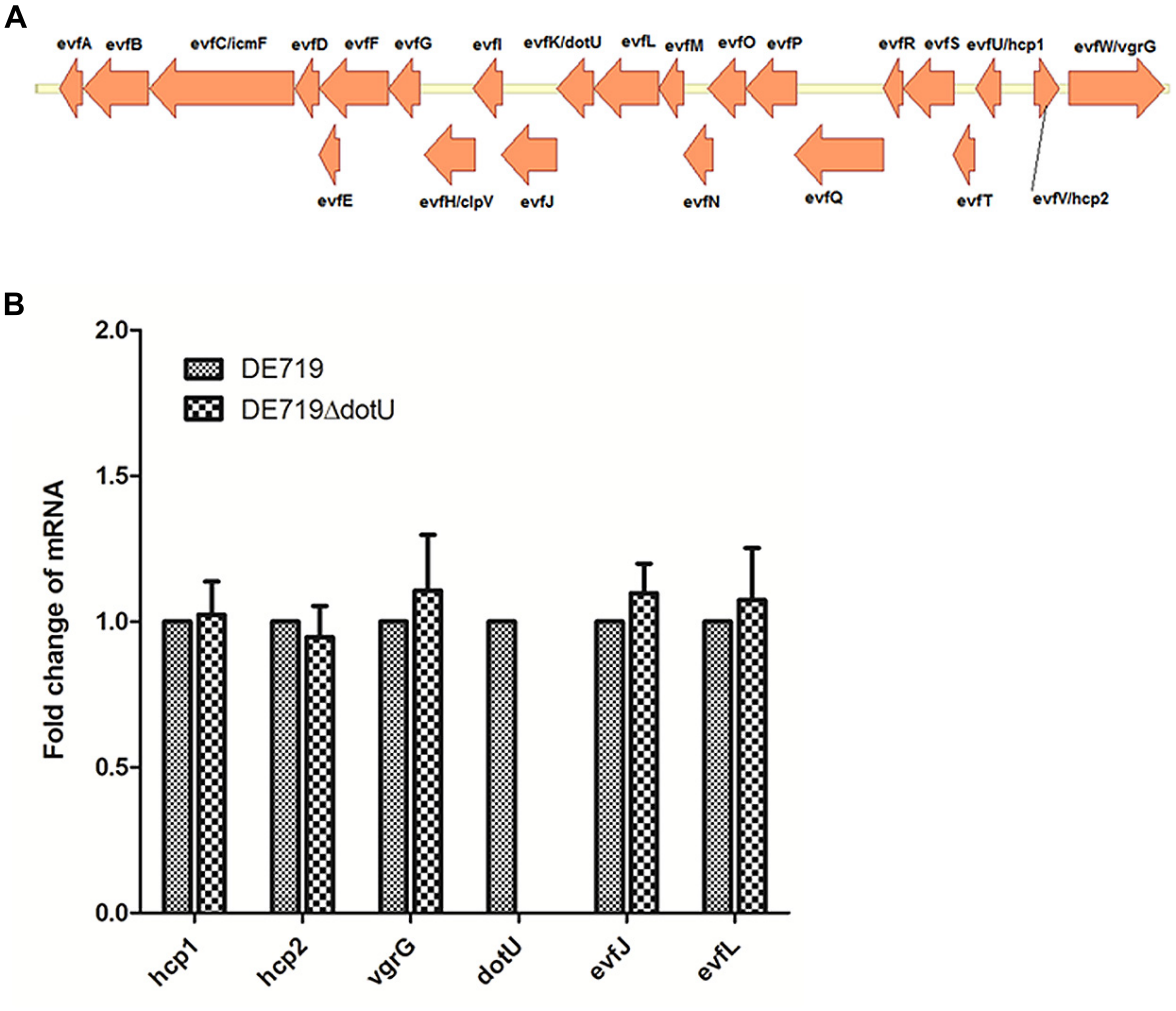
**(A)** Schematic diagram of the genetic organization of APEC DE719 T6SS2 gene clusters. The T6SS2 whole-gene cluster of ORFs with different direction is shown. **(B)** Quantification of T6SS2 core genes expression. Transcript levels of *hcp1*, *hcp2*, *vgrG*, *dotU*, *evfJ,* and *evfL* in DE719 and DE719ΔdotU analyzed by qRT-PCR. Data were normalized to the housekeeping gene *dnaE* and shown as fold changes.

Genome sequence analysis showed that ORF order and component genes of T6SS2 in APEC DE719 were the same as in NMEC RS218 (**Figure 1A**). To determine whether *dotU* mutation had a polar effect on upstream or downstream genes, transcription levels of *hcp1*, *hcp2*, *vgrG*, *evfJ,* and *evfL* of the DE719 and DE719ΔdotU were analyzed by qRT-PCR. Deletion of the *dotU* gene had no influence on expression of the upstream and downstream genes in T6SS2 (**Figure 1B**).

### DotU INDUCTION AND ANTI-DotU ANTIBODY PRODUCTION IN DUCKS

**FIGURE 2:**
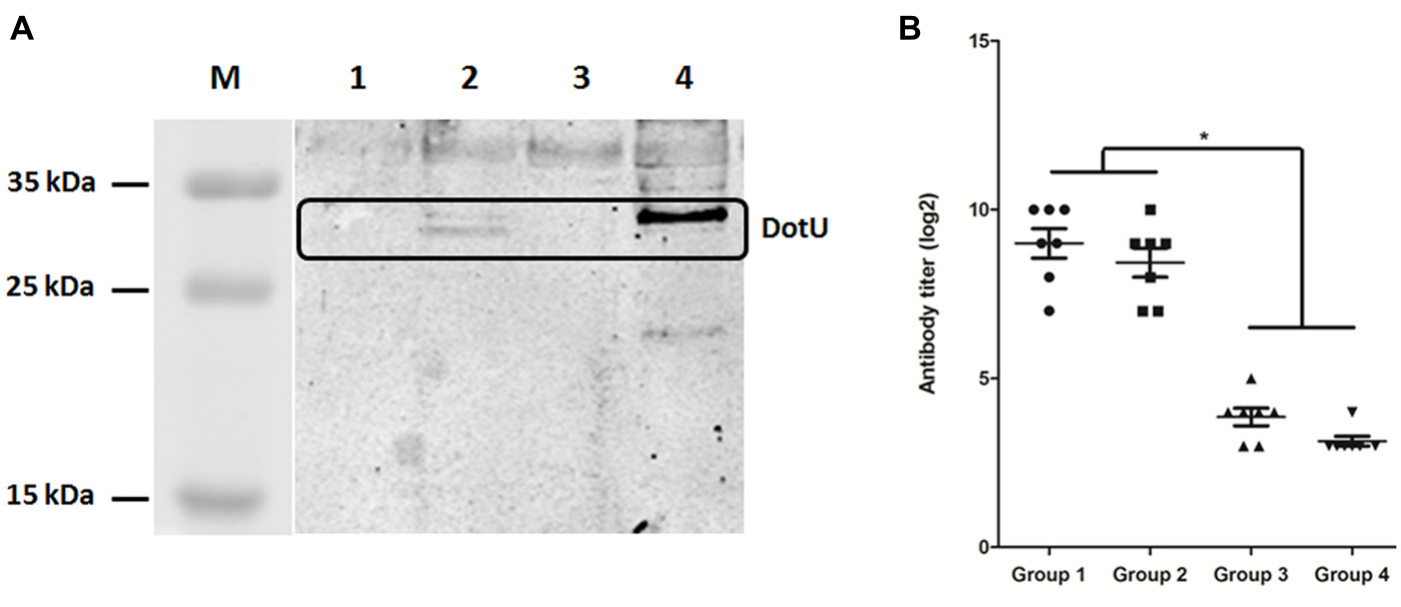
**DotU expression and anti-DotU antibody production. (A)** Western blotting for DotU expression using duck anti-DotU. Lane M, prestained protein marker; Lane 1, DE719ΔdotU cell lysate; Lane 2, DE719CΔdotU cell lysate; Lane 3, DE719 cell lysate; Lane 4, purified recombinant DotU. **(B)** ELISA for anti-DotU titers in duck sera. Group 1, APEC DE719 infected duck sera; Group 2, APEC DE719 infected duck sera pre-adsorbed with DE719ΔdotU; Group 3, Duck sera induced by inactivated APEC DE719 immunization; Group 4, Negative sera from ducks inoculated with PBS. *p < 0.05.

Analysis by qRT-PCR revealed considerably lower transcription of *dotU* in LB-cultured DE719 compared to the housekeeping gene *dnaE* (ΔCt = 4.77 ± 0.77, Ct*_dnaE_* = 26.14 ± 1.49, Ct*_dotU_* = 30.91 ± 0.79). Thus, western blotting was performed with anti-DotU serum, which showed that expected protein bands of DotU were detected for complementation strain DE719CΔdotU. However, there were no detectable bands for wild-type and mutant strains (**Figure 2A**). To further investigate DotU expression *in vivo*, ELISA was used to measure DotU antibody titers in ducks. Anti-DotU titers in APEC DE719-infected ducks was 2^9^ on average. In addition, pre-adsorbed sera by DE719ΔdotU cells produced similar anti-DotU titer to APEC DE719 infected duck sera, suggesting the antibody is specific to the DotU, not a background reading. Contrastively, duck sera induced by inactived APEC DE719 immunization yielded very low anti-DotU titer, which is similar to the negative sera (**Figure 2B**). Thus, the results showed that DotU was highly induced during APEC infection.

### DotU IS INVOLVED IN BACTERIAL RESISTANCE TO NORMAL DUCK SERUM KILLING

**FIGURE 3:**
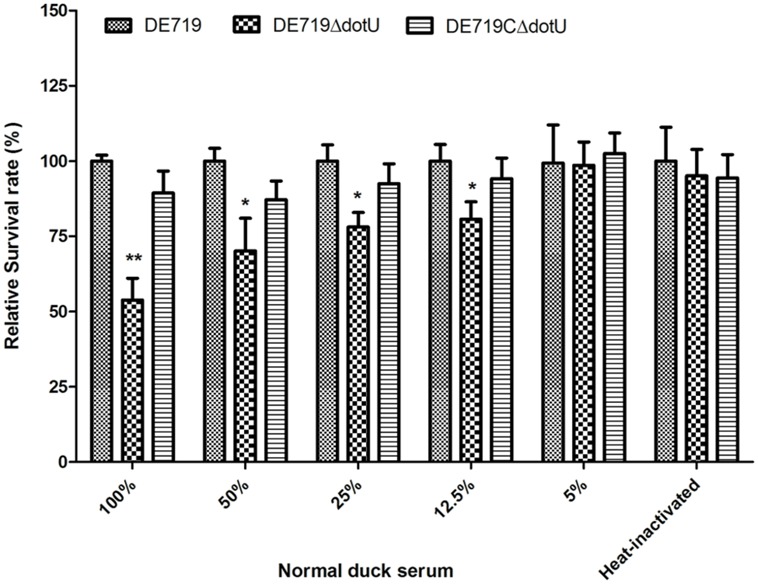
**Bacterial resistance to normal duck serum.** Bacteria were incubated with the normal duck serum at different dilution at 37°C, and CFUs were enumerated after 30 min treatment. The data revealed that DE719ΔdotU showed significant reduction of resistance. **p < 0.01; *p < 0.05.

Avian pathogenic *Escherichia coli* causes typical avian colibacillosis, with bacteria invading air sacs, blood, pericardial fluid, and the typical fibrinous lesions. Thus, resistance to serum is associated with APEC pathogenicity. Bactericidal assays revealed that the mutant strain DE719ΔdotU had lower resistance to normal duck serum than the wild-type strain DE719. Resistance was restored in the complementation strain (**Figure 3**). These results indicated that DotU was involved in bacterial serum resistance.

### DotU FACILITATES APEC ADHESION TO DF-1 CELLS

**FIGURE 4:**
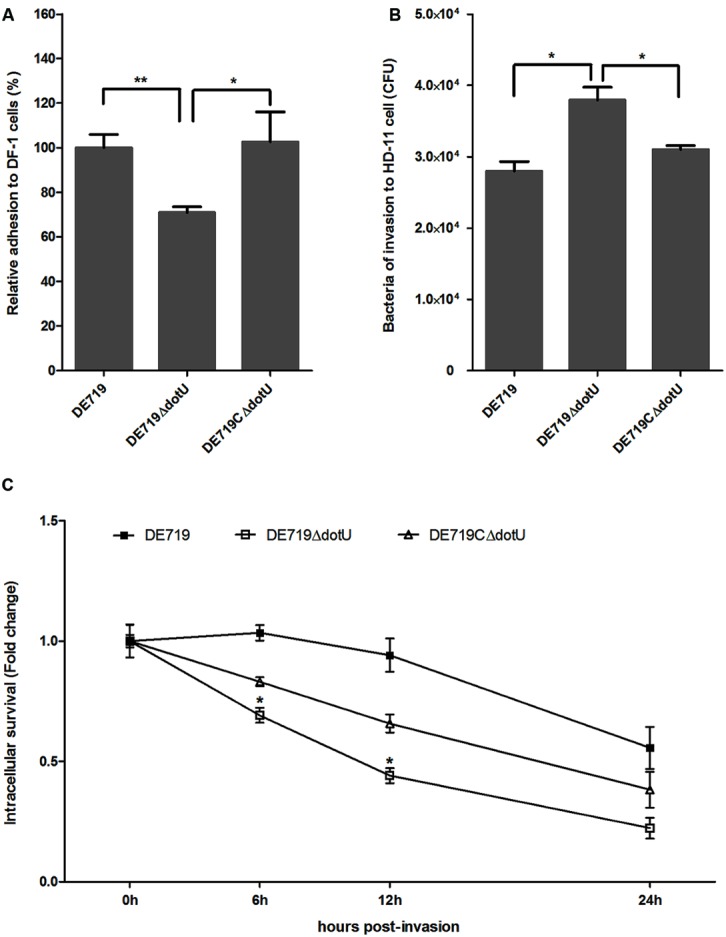
**Bacterial adhesion, invasion, and intracellular survival assays. (A)** Adhesion assays performed on DF-1 cells. Values are average of three independent experiments. Error bars indicate standard deviations. One-way ANOVA was performed for statistical significance. **p < 0.01; *p < 0.05. **(B)** Invasion assays with chicken macrophage HD-11 cells. Values are average of three independent experiments. Error bars indicate standard deviations. One-way ANOVA was performed for significance. *p < 0.05. **(C)** Intracellular survival in chicken macrophage HD-11 cells, expressed as fold change in bacterial number at 6, 12, and 24 h relative to initial invasion. *p < 0.05, compared with DE719.

DotU effects on bacterial adhesion and invasion to avian cell lines were determined. DF-1 cells were infected with DE719, DE719ΔdotU, and the complementation strain DE719CΔdotU. Numbers of bacteria adhering to and invading DF-1 cells were determined. No significant differences were observed among the bacterial strains for invasion capacity, indicating that DotU did not affect APEC invasion of DF-1 cells (data not shown). However, adherence of the mutant strain DE719ΔdotU was significantly reduced compared with the wild-type strain DE719 (*p* < 0.01; **Figure 4A**). Adhesion capacity was restored in the complementation strain DE719CΔdotU. These results suggested that DotU plays a role on adherence of APEC to DF-1 cells.

### DotU CONTRIBUTES TO INTRACELLULAR SURVIVAL IN MACROPHAGES

Systemic dissemination is dependent on survival within phagocytic cells. Therefore, DotU involvement in intracellular survival and replication was assessed at 0, 6, 12, and 24 h post-invasion of HD-11 cells. Compared to DF-1, the mutant strain DE719ΔdotU exhibited significantly increased invasiveness of macrophage HD-11 cells compared to wild-type and the complementation strain (*p* < 0.05; **Figure 4B**). However, the wild-type strain had a higher intracellular survival rate than the mutant strain DE719ΔdotU at all time points tested. Partial complementation for intracellular survival capacity was observed for strain DE719CΔdotU (**Figure 4C**). Thus, DotU was essential for APEC intramacrophage survival.

### DotU AND T6SS2 AFFECT BACTERIAL COLONIZATION AND SURVIVAL DURING INFECTION *IN VIVO*

**FIGURE 5:**
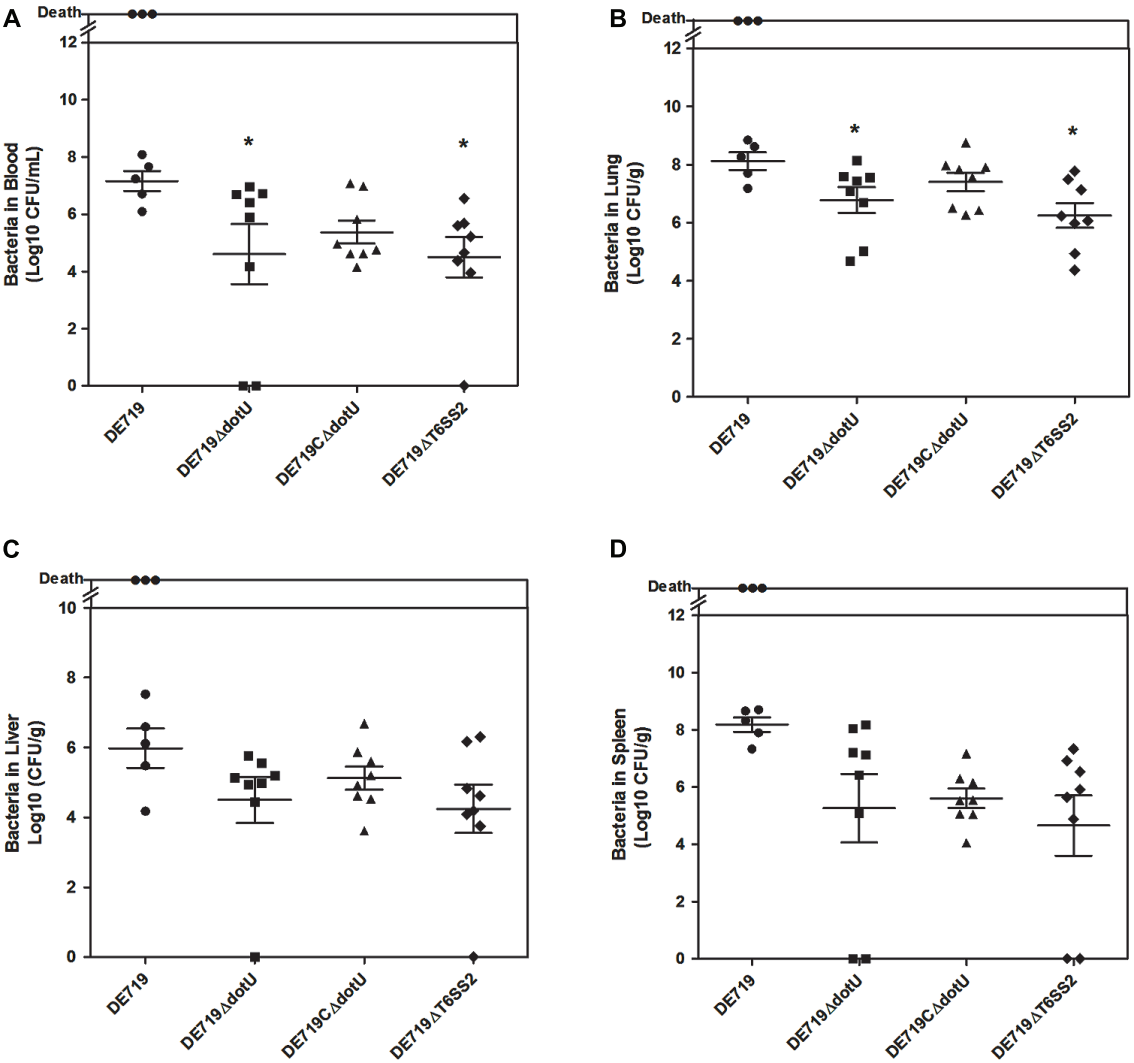
**Animal systemic infection experiments.** Groups of eight 7-day-old ducks were intratracheally infected with 10^8^ CFU bacteria. Bacteria were recovered from blood **(A)**, lungs **(B)**, livers **(C)**, and spleens **(D)** at 24 h post-infection. *p < 0.05 compared with DE719. Non-parametric Mann–Whitney U-test was carried out for statistical significance.

To determine the role of DotU *in vivo* infection, ducks were infected intratracheally with DE719, DE719ΔdotU, DE719CΔdotU, and DE719ΔT6SS2. Bacterial loads in blood, lung, liver, and spleen were investigated at 24 h post-infection. Colonization of mutant strains DE719ΔdotU and DE719ΔT6SS2 in the blood and lung were significantly reduced compared to wild-type DE719 (*p* < 0.05). DE719CΔdotU had recovered bacterial colonization capacity (**Figures 5A,B**). Bacterial loads in the liver and spleen of infected ducks were not significantly different among DE719, DE719ΔdotU, DE719CΔdotU, and DE719ΔT6SS2 (*p*> 0.05; **Figures 5C,D**). These results indicated that DotU and T6SS2 were involved in colonization and survival during infection *in vivo*.

### DELETION OF DotU AND T6SS2 ATTENUATES APEC STRAIN DE719 VIRULENCE *IN VIVO*

**FIGURE 6:**
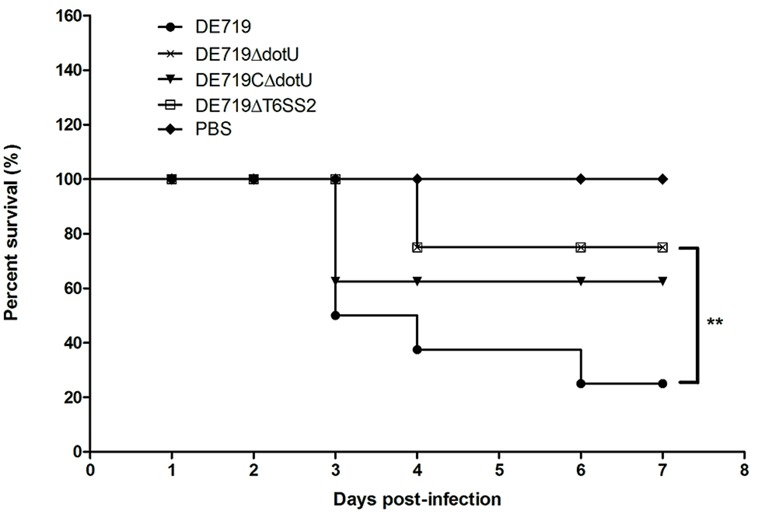
**Determination of bacterial virulence.** 7-day-old ducks were inoculated intratracheally with DE719, DE719ΔdotU, DE719CΔdotU, or DE719ΔT6SS2 suspensions at 10^7^ colony-forming units (CFUs). Negative controls were injected with PBS. Survival was monitored until 7 days post-infection. **p < 0.01, compared with DE719.

To investigate if DotU or T6SS2 affected bacterial virulence, groups of eight ducks were infected with bacteria at 1 × 10^7^ CFU. Mortality was 75% (6/8) for infection with DE719, 12.5% (1/8) for DE719ΔdotU, 37.5% (3/8) for DE719CΔdotU, and 12.5% (1/8) for DE719ΔT6SS2 (**Figure 6**). These results indicated that inactivation of *dotU* or T6SS2 locus attenuated virulence in ducks. Virulence was partly restored in the complementation strain. These results provided evidence that DotU and T6SS2 were important virulence factors in APEC strains.

### DotU WAS INVOLVED IN THE SECRETION OF Hcp1 BY T6SS

**FIGURE 7:**
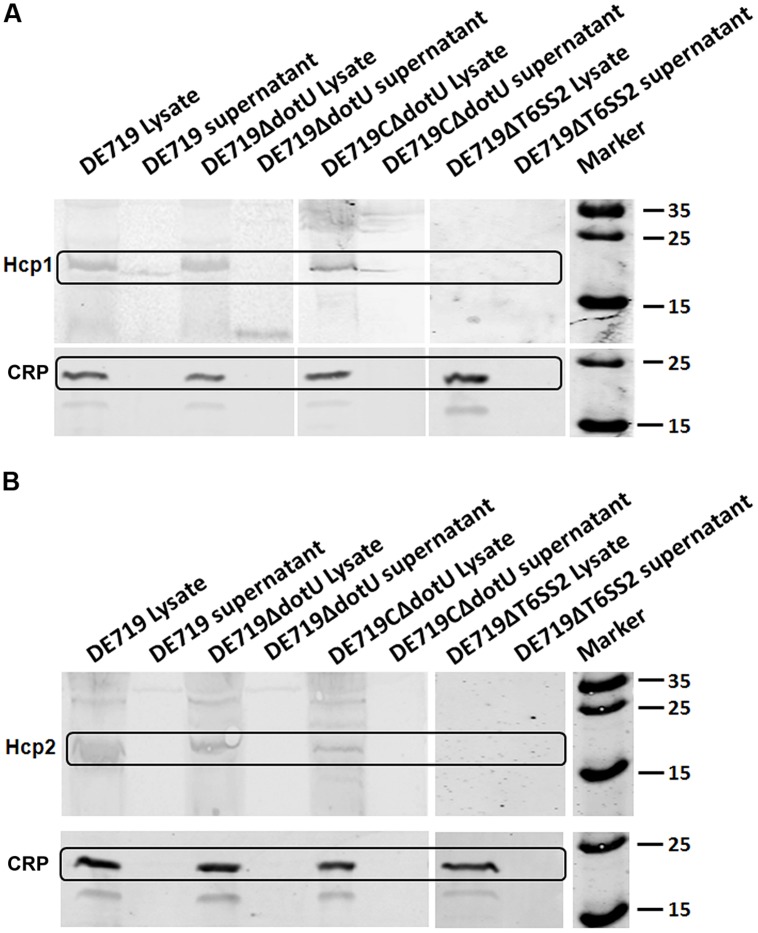
**DotU was essential for Hcp1 secretion by T6SS2 in APEC. (A)** Hcp1 secretion in APEC supernatants by western blot using anti-Hcp1. Hcp1 protein was detected at the expected size in culture supernatants of DE719 and DE719CΔdotU. No Hcp1 secretion was detected in supernatants of DE719ΔdotU and DE719ΔT6SS2. **(B)** No Hcp2 was detectable in supernatants of DE719 and DE719CΔdotU or lysates and supernatant of DE719ΔT6SS2. CRP was the cytoplasmic protein marker.

Hcp family proteins are secreted via a T6SS-dependent pathway in several bacteria and are detected in bacterial culture supernatants (Mougous et al., 2006; Aschtgen et al., 2010; Mulder et al., 2012; Zhou et al., 2012). Culture supernatants and APEC DE719, DE719ΔdotU, DE719CΔdotU, and DE719ΔT6SS2 cells were tested for Hcp1 and Hcp2 by western blotting using corresponding polyclonal antisera. Hcp1 was detected in bacterial lysates of APEC DE719, DE719ΔdotU, DE719CΔdotU, and the culture supernatants of APEC DE719 and DE719CΔdotU. No Hcp1 was detected in culture supernatants of DE719ΔdotU or supernatants or lysates of DE719ΔT6SS2, demonstrating that DotU contributed to secretion of Hcp1 by T6SS2 in APEC DE719 (**Figure 7A**). Hcp2 protein was detected only in lysates of APEC DE719, DE719ΔdotU and DE719CΔdotU. No Hcp2 was detected in supernatants or lysates of DE719ΔT6SS2 (**Figure 7B**), which is similar to previous reports of NMEC strain RS218 (Zhou et al., 2012).

### DETERMINATION OF THE CYTOKINE EXPRESSION IN APEC INFECTED HD-11 CELLS

**FIGURE 8:**
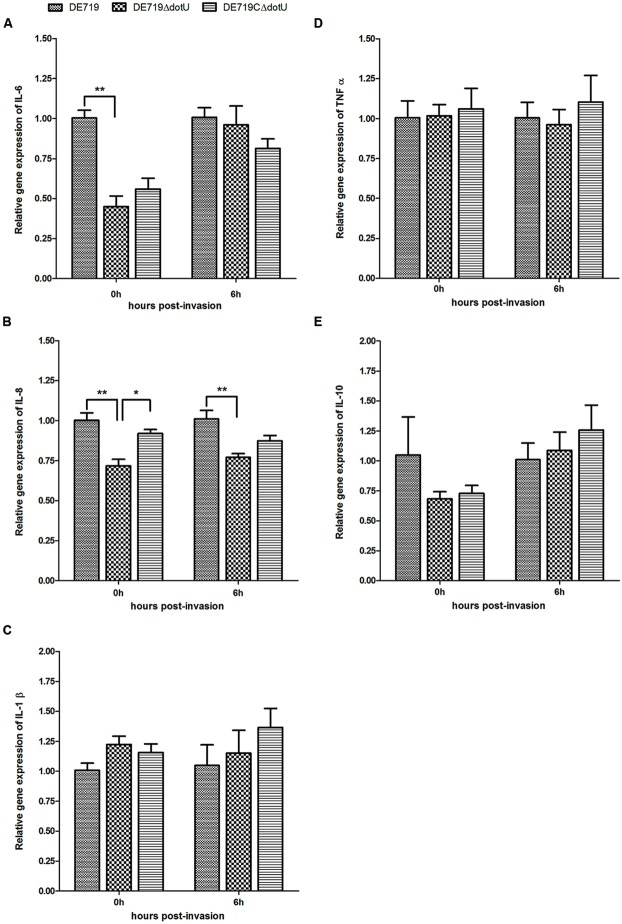
**Cytokine expression in the HD-11 cells infected with APEC bacteria.** Expression of IL-6 **(A)**, IL-8 **(B)**, IL-1β **(C)**, TNFα **(D)** and IL-10 **(E)** in infected HD-11 cells was analyzed by RT-PCR, normalized to β-actin gene. Samples were calibrated to gene expression in DE719 infected HD-11 cells. Expression of IL-6 and IL-8 was significantly downregulated by 2.2-fold (IL-6, 0 h post-invasion), 1.4-fold (IL-8, 0 h post-invasion), and 1.3-fold (IL-8, 6 h post-invasion) in cells treated with DE719ΔdotU compared with DE719. **p < 0.01; *p < 0.05. Statistical significance analysis was performed by two-way ANOVA.

To assess the effects of *dotU* deletion on macrophage cytokine expression, HD-11 macrophages were infected with DE719, DE719ΔdotU, or DE719CΔdotU. Expression of IL-1β, IL-6, IL-8, IL-10, and TNFα in infected cells was analyzed at 0 h and 6 h post-invasion by qRT-PCR. IL-6 and IL-8 expression was downregulated in cells treated with DE719ΔdotU compared with cells treated with DE719. Differences in the expression of IL-6 at 0 h, IL-8 at 0 h and 6 h were significant (*p* < 0.05 or *p* < 0.01). Levels of IL-6 and IL-8 were partially restored by expressing DotU in *trans* (**Figure 8**). Levels of IL-1β, IL-10, and TNFα were not significantly different (*p*> 0.05).

## DISCUSSION

Recently, three distinct T6SS loci were discovered in the APEC genome, distributed as 14.62% T6SS1, 2.33% T6SS2, and 0.85% T6SS3 in the APEC collections. Comprehensive analysis showed that more than 85% of T6SSs loci-containing APEC strains belong to the virulent phylogenetic groups D and B2 (Johnson et al., 2006), indicating that T6SSs might contribute to APEC pathogenicity (Ma et al., 2013). Several T6SS proteins including Hcp, VgrG, IcmF, and DotU are important for bacterial pathogenesis. Previous studies showed that APEC T6SS1 is involved in colonization and proliferation in systemic infections and T6SS2 is responsible for intramacrophage survival, cytokine and chemokine release, and host cell apoptosis. However, the function of most T6SS proteins remains unknown (Filloux, 2009; Pukatzki et al., 2009; Silverman et al., 2012).

Our study showed that APEC *dotU* was expressed at low levels in LB culture. However, ELISA showed high levels of DotU antibodies in infected ducks, suggesting that APEC DotU interacted with host cells during APEC infection. We examined the contribution of DotU and T6SS2 to APEC pathogenicity. APEC *dotU* and T6SS2 locus mutant strains and an APEC *dotU* complementation strain were constructed. Animal experiments showed that the virulence of *dotU* and T6SS2 mutant strains was attenuated compared with the wild-type strain DE719. The complemented strain had recovered virulence. The *dotU* and T6SS2 mutant strains did not exhibits growth defects. Thus, we concluded that DotU and T6SS2 were necessary for full virulence of APEC DE719. Colonization is a crucial step for bacterial pathogenesis (Finlay and Falkow, 1997). Bacterial infection studies *in vitro* and *in vivo* indicated that loss of DotU or T6SS2 resulted in significantly reduced colonization of DF-1 cells and duck blood and lungs compared to wild-type strain. These results might indicate the reason for attenuated virulence of the mutant strain DE719ΔdotU.

Avian pathogenic *Escherichia coli* infects poultry by initial respiratory tract colonization followed by systemic spread. Serum resistance is an important virulence parameter for APEC infection. Resistance to the bactericidal effects of serum and the capacity of APEC strains to cause septicemia and mortality are correlated (La Ragione and Woodward, 2002; Mellata et al., 2003). Resistance to serum and environmental stress and survival within macrophages are advantages to APEC infection. We examined the survival of APEC in normal duck serum and macrophages. Bactericidal assays demonstrated that resistance to normal duck serum was impaired in the mutant strain DE719ΔdotU (**Figure 3**). DE719ΔdotU showed a significantly reduced survival rate in macrophage HD-11 cells (**Figure 4**). Resistance defects might be a reason for the reduced bacterial survival of mutant strain DE719ΔdotU in hosts.

Hemolysin-coregulated protein family proteins are secreted via a T6SS-dependent pathway and act in bacterial interaction with host cells (Mougous et al., 2006; Aschtgen et al., 2010; Mulder et al., 2012; Zhou et al., 2012). Secretion of the Hcp family proteins Hcp1 and Hcp2 from DE719 and DE719ΔdotU, DE719ΔT6SS2 was determined. Hcp1 but not Hcp2 was secreted by APEC T6SS2, consistent with NMEC RS218 results (Zhou et al., 2012). DotU was essential for secretion of Hcp1 by T6SS2 in APEC DE719. DotU was interacts with IcmF and prevents degradation of other proteins through an unknown mechanism (Sexton et al., 2004; Zheng and Leung, 2007; Ma et al., 2009; Broms et al., 2012). Deletion of DotU might lead to defects in T6SS2 structural integrity and effector protein Hcp1 secretion, although this hypothesis needs further investigation. To eluciate Hcp1 functions, we generated the mutant strain DE719Δhcp1, which showed adhesion and invasion capacities similar to the wild-type strain DE719 (data not shown), suggesting that Hcp1 did not affect bacterial adhesion and invasion, consistent with a previous study (Zhou et al., 2012). Thus, the reduced colonization was due to DotU deletion.

Type VI secretion systems induces cytokine release, actin cytoskeleton rearrangement, and apoptosis in HBMECs. These strategies are exploited by pathogenic bacteria for survival or spread in the host (Zhou et al., 2012). However, our study found no detectable difference in apoptosis in cells stimulated with DE719 or the mutant strain DE719ΔdotU (data no shown). The mutant strain DE719ΔdotU induced lower levels of IL-6 and IL-8 gene expression than DE719 (**Figure 8**), which might be due to the abolition of Hcp1 secretion. IL-6 and IL-8 are important inflammatory mediators in inflammation and leukocyte recruitment and contribute to host immunity or pathogenesis (Zhou et al., 2012). Our data suggested that DotU might affect cytokine production regulating inflammation initiation and cell recruitment, thereby affecting downstream immune response pathways.

**FIGURE 9:**
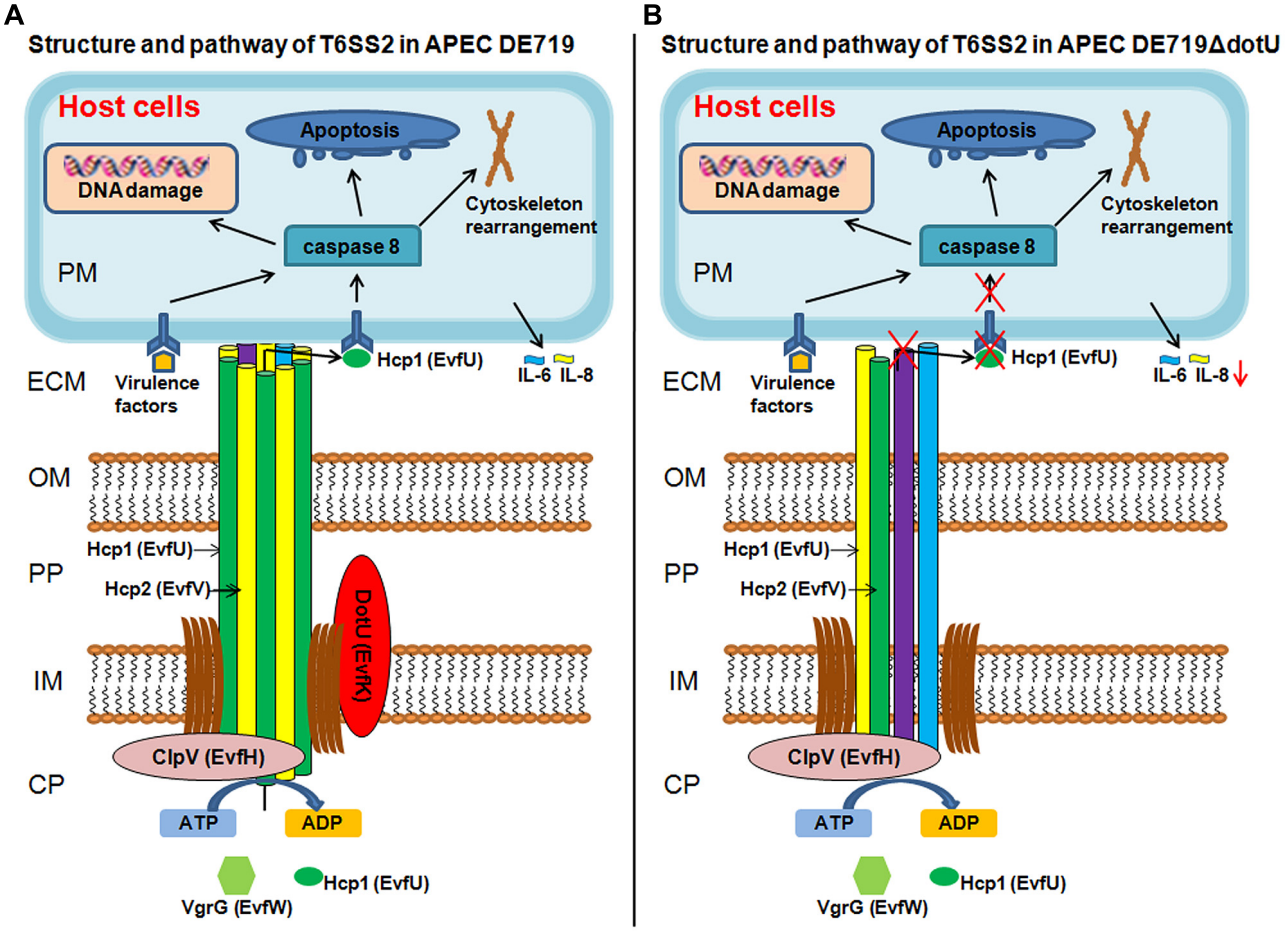
**Schematic T6SS2 structure and pathway in APEC *dotU* mutant. (A)** T6SS2 structure and pathway in APEC strains. The channel through the bacterial periplasm is Hcp1, Hcp2, and other proteins that transport effector protein Hcp1 to the ECM. Hcp1 could be recognized by a specific receptor of host cells, leading to cytoskeleton rearrangement, apoptosis, and cytokine release. As the core component, DotU stabilizes the secretion machinery. **(B)** Deletion of DotU might lead to defective T6SS2 structure, abolishing secretion of effector protein Hcp1. The defective needle structure might also affect cell aggregation, resistance to normal duck serum, intramacrophage survival and colonization *in vitro* and *in vivo*. CP, bacterial cytoplasm; IM, bacterial inner membrane; PP, bacterial periplasm; OM, bacterial outer membrane; ECM, extracellular milieu; and PM, host cell plasma membrane.

In summary, we propose a potential model for DotU in APEC infection (**Figure 9**) showing the structure and pathway of T6SS2 in wild-type strain DE719 and mutant strain DE719ΔdotU. The needle structure of APEC T6SS2 is composed of Hcp1, Hcp2, and other proteins and transports effector protein Hcp1 to the extracellular milieu. Secreted Hcp1 is recognized by a specific receptor of host cells, leading to events including cytoskeleton rearrangement, apoptosis, and cytokine release. The needle structure might increase bacterial capacities of cell aggregation, resistance to normal duck serum, intramacrophage survival and colonization *in vitro* and *in vivo*, which are essential for bacterial pathogenesis. Our results suggested that DotU was highly expressed during APEC infection. Deletion of DotU affected Hcp1 secretion and involved in the integrity of the T6SS2 apparatus, resulting in defective function and virulence.

## Conflict of Interest Statement

The authors declare that the research was conducted in the absence of any commercial or financial relationships that could be construed as a potential conflict of interest.
